# Impact of COVID-19 on Timeliness of Receiving Systemic Therapy for Patients Diagnosed with Lung Cancer

**DOI:** 10.4103/ejcrp.ejcrp-d-25-00002

**Published:** 2025-08-14

**Authors:** Mahesh Maiyani, Kris Wain, Nikki M. Carroll, Jennifer Eisenstein, Brian Hixon, Robert T. Greenlee, Christine Neslund-Dudas, Caryn Oshiro, Debra P. Ritzwoller

**Affiliations:** 1 Institute for Health Research, Kaiser Permanente Colorado, Aurora, CO, USA; 2 Department of Oncology, Colorado Permanente Medical Group, Kaiser Permanente Colorado, Denver, CO, USA; 3 Marshfield Clinic Research Institute, Marshfield Clinic Health System, Marshfield, WI, USA; 4 Department of Public Health Science, Henry Ford Health and Henry Ford Cancer Institute, Detroit, MI, USA; 5 Center for Integrated Health Care Research, Kaiser Permanente Hawaii, Honolulu, HI, USA

**Keywords:** COVID-19, delay in treatment, lung cancer, systemic therapy, time to treatment

## Abstract

**Background:**

During the COVID-19 pandemic, patients newly diagnosed with lung cancer faced potential delays in accessing timely cancer treatment. Little information exists on how COVID-19 impacted the timeliness of receiving a first course of systemic therapy after a lung cancer diagnosis.

**Materials and Methods:**

Data from the Population-based Research to Optimize the Screening Process-Lung Consortium were used to identify patients diagnosed with Stage I to IV lung cancer between January 1, 2018 and September 30, 2021, for this retrospective cohort study. The patients were categorized into three groups based on the date of diagnosis: pre-COVID-19 (January 1, 2018–March 14, 2020), early COVID-19 (March 15, 2020–June 30, 2020), and late COVID-19 (July 1, 2020–September 30, 2021). We explored changes in the time from cancer diagnosis to initiation of the first course of systemic therapy using interrupted time series (ITS) analysis across the pre-, early, and late COVID-19 time periods.

**Results:**

We identified 810 patients with lung cancer who received a first course of systemic therapy during the study period. The average number of patients diagnosed with lung cancer per month decreased from 17.0 in the pre-COVID-19 period to 15.3 in the early COVID-19 period and then increased to 19.3 in the late COVID-19 period. ITS models estimated a 9.6-day increase (95% confidence interval: 4.8, 14.5; *P* < 0.01) from lung cancer diagnosis to treatment initiation at the start of the early COVID-19 period. The time from lung cancer diagnosis to treatment initiation returned to pre-COVID-19 levels by the start of the late COVID-19 period.

**Conclusion:**

Our findings indicate a delay in initiating systemic therapy among patients with lung cancer during the early COVID-19 period compared to the pre- and late COVID-19 periods; however, the time to initiating treatment recovered quickly.

## Introduction

The American Cancer Society estimates that 234,580 new lung cancer cases will be diagnosed in 2024, resulting in 125,070 deaths.^[1]^ During the COVID-19 pandemic, healthcare systems faced an increased demand for services while also implementing government-mandated precautions, which restricted access to lung cancer treatment.^[2,3]^ In addition, many cancer centers faced shortages of systemic therapy drugs during the COVID-19 shutdown, potentially exacerbating delays in lung cancer treatment.^[4–6]^ Given the aggressive progression of lung cancer, delayed treatment can be less effective, leading to poorer survival and quality of life.^[7–10]^ Moreover, studies have shown that the COVID-19 pandemic reduced participation in lung cancer screening programs, leading to more advanced-stage diagnoses that are more difficult to treat.^[11,12]^ A CHEST expert panel of pulmonologists, thoracic surgeons, and radiologists recommended delaying lung cancer screening, surveillance, and treatment for early-stage cancer due to the added risks from potential COVID-19 exposure and the need for resource reallocation.^[13]^ Understanding whether healthcare systems can swiftly recover from unanticipated delays in cancer treatment is essential to help decision-makers efficiently allocate resources during future supply chain disruptions. Guidelines from professional societies can assist clinicians to prioritize patients with higher-risk disease to prevent treatment delays and propose alternatives such as initiating systemic therapy first, to allow for the postponement of surgical treatment.^[14–16]^

Existing studies have shown the impact of COVID-19 pandemic on lung cancer screening, diagnosis, and treatment delays.^[10,17–20]^ These studies found delays in the diagnosis, an increase in the proportion of patients with advanced non-small cell lung cancer, delays in treatment, poor survival, and higher stage of lung cancer. However, many of these studies were limited by short follow-up periods, which prevented them from examining whether delays extended into the COVID-19 recovery period. Other studies have relied on patient-reported data, which may be influenced by recall bias, or examined treatment delay as a binary outcome and were unable to assess the duration of the delay.^[18,19,21–23]^ Limited research has investigated the length of the delay in initiating systemic therapy and whether these delays continued beyond the COVID-19 shutdown period and into the recovery phase.

Therefore, the aim of this study was to assess the impact of the COVID-19 pandemic on the timeliness of receiving a first course of systemic therapy among patients newly diagnosed with lung cancer during pre-, early, and late COVID-19 time periods. We analyzed the number of days between diagnosis and treatment initiation for each time period and assessed how quickly the healthcare systems returned to pre-COVID-19 rates by analyzing data through December 2021. Understanding how healthcare systems managed delays in cancer treatment during the COVID-19 shutdown can help healthcare providers and oncologists proactively enhance treatment protocols that can adapt to challenges when recommended care is disrupted by supply chain issues, staff shortages, or other unforeseen disruptions.

## Materials and Methods

### Study setting and data source

This retrospective cohort study was conducted using data from the Population-based Research to Optimize the Screening Process (PROSPR)-Lung Consortium, a collaboration of five diverse, integrated healthcare systems, including Kaiser Permanente Colorado (KPCO), Kaiser Permanente Hawaii (KPHI), Henry Ford Health (HFH), Marshfield Clinic Health System (MCHS), and University of Pennsylvania Health System (UPHS), where patients receive both primary and oncology care services.^[24]^ The PROSPR-Lung Consortium developed a standardized common data model derived from electronic health record data on patient demographics, diagnoses, cancer registry variables, and census-based measures of socioeconomic status on patients aged 35–89 years who were engaged with these healthcare systems between January 1, 2010, and December 31, 2021. Cancer registry data were collected in accordance with the standards of the North American Association of Central Cancer Registries^[25]^ by certified tumor registrars and included diagnosis dates, cancer stage, tumor characteristics, and first-course therapy. Data from the following four sites were included in our analyses: KPCO, KPHI, HFH, and MCHS. UPHS was excluded due to the incompleteness of infused therapy data. This study followed the STROBE guidelines for reporting cohort studies.^[26]^

This study adhered to the principles of the Declaration of Helsinki (2013 revision) and received approval from the Interregional Review Board of Kaiser Permanente (Approval No. CO-17–2466_02, approval date: 09/05/2018), with a waiver of individual consent granted for this retrospective analysis.

### Study population

The study population for this analysis included PROSPR members diagnosed with American Joint Committee on Cancer (AJCC) Stage I to IV lung cancer between January 1, 2018, and September 30, 2021. Demographic, clinical, and tumor characteristics were obtained from the PROSPR-Lung common data model. All of the included individuals were continuously engaged with the healthcare system at the time of lung cancer diagnosis and had consistent access to care. In addition, all of the included patients received systemic therapy as their first course of treatment within 180 days of the diagnosis. As systemic therapy received after 180 days may not be related to the initial lung cancer diagnosis (e.g., could signal the progression of disease or recurrence), these patients were excluded from the study. Engagement was defined as either continuous enrollment in the health plan for capitated healthcare systems or at least one primary care encounter for non-capitated systems. Patients with stage 0, missing or unknown stage, those who did not receive any first-course treatment, and those who received surgery or radiation therapy as their first course of treatment were also excluded. We further excluded patients who died within 3 months of their lung cancer diagnosis and those diagnosed with lung cancer before January 1, 2018.

### Exposure and outcomes

The patients were categorized into three mutually exclusive time period groups: (1) pre-COVID-19, which included patients diagnosed between January 1, 2018, and March 14, 2020 as baseline; (2) early COVID-19, which included patients diagnosed between March 15, 2020, and June 30, 2020, corresponding to the shutdown period based on the timeline established by the Centers for Disease Control and Prevention;^[27,28]^ and (3) late COVID-19, which included patients diagnosed between July 1, 2020, and September 30, 2021, when the participating healthcare systems began restoring access to services.^[27]^

Our primary outcome was the timeliness of treatment, defined as the number of days from the lung cancer diagnosis to the initiation of systemic therapy. Systemic therapy was defined as any cytotoxic chemotherapy, targeted therapy, immunotherapy, or monoclonal antibody therapy. Treatment data from the tumor registry were used to identify the type and date of systemic therapy initiation.^[29]^

### Descriptive characteristics and stratification variables

All patient-level data were collected at the time of diagnosis: age, sex, race, ethnicity, and AJCC stage. Age was categorized into three groups: 35–64, 65–74, and 75–89 years. Self-reported race and ethnicity were categorized into White, Black, Hispanic, Asian, Native Hawaiian/Pacific Islander, and Other races. Due to the small number of patients identifying as American Indian and Alaska Native, and multiracial, these patients and those with missing data for race were combined into an unknown/other race category. Smoking status was determined as that recorded closest to the diagnosis and was categorized as patients who currently smoke, patients who formerly smoked, and patients whose smoking history was unknown or missing. The stage of lung cancer at the time of diagnosis was included as a binary variable: Stages I, II, and IIIA were considered early stage, while Stages IIIB and Stage IV were considered late stage.^[30]^ The healthcare system was examined as a categorical variable to adjust for site-level differences in cancer treatment during each COVID-19 period. We used the Deyo *et al*. adaptation of the Charlson Comorbidity Index to assess the comorbidity burden.^[31]^ We used the state-level YOST Index to measure socioeconomic status at the census tract level. The YOST Index was classified into quintiles, with the 1^st^ quintile representing the most deprived areas and the 5^th^ quintile representing the most affluent areas.^[32]^

To address immortal time bias and to ensure adequate time for initiating systemic therapy, we excluded patients (*n* = 977) who disenrolled or died within 3 months following their lung cancer diagnosis. Among those who died within 3 months of diagnosis, 64% (*n* = 370/582) did not receive any treatment, and 79% (*n* = 460/582) were diagnosed with late-stage lung cancer. We did not have the treatment information for 395 patients who were disenrolled/not engaged with the healthcare systems.

### Statistical analysis

Baseline demographic characteristics were compared between patients in each time period using Chi-squared tests. We used single-group interrupted time series (ITS) analysis to estimate the number of days between lung cancer diagnosis and initiation of the first course of systemic therapy. Our ITS model employed ordinary least squares regression with Newey–West standard errors to account for the potential of autocorrelation and heteroskedasticity in the residuals.^[33]^ The Cumby–Huizinga test was used to check for autocorrelation and confirm that the ITS model accurately fit the autocorrelation structure and error distribution.^[33]^ To assess the impact of COVID-19 on treatment delays, we reported the estimated mean number of days from diagnosis to treatment at the start of each time period.

To better understand trends within each time period, we estimated a monthly linear time trend using the start of each period as a reference point to quantify the change in the number of days from diagnosis to treatment. We analyzed 27 months of data from the pre-COVID-19 period to establish a baseline monthly trend. The early COVID-19 period included 4 months of data and enabled us to estimate the initial impact of the shutdown on the timeliness of receiving systemic therapy. The late COVID-19 period included 19 months of data, which allowed us to assess how quickly the healthcare systems returned to pre-COVID-19 rates.

Because ITS analysis does not allow for adjustment of individual-level characteristics, we estimated stratified models to gain insights into factors that might explain differences between time periods.^[12,33,34]^ We re-estimated our ITS model stratified by stage, race, sex, and age at diagnosis. Due to a significant reduction in sample size in our stratified models, which limited precision, we chose to estimate the average time from diagnosis to initiation of systemic therapy for each of the three time periods and did not include a monthly linear time trend. Interaction terms between each subgroup and COVID-19 time period were examined to assess whether their combined effects influenced the time to treatment. To evaluate the robustness of our findings, we generated a multivariable linear regression model, adjusting for COVID-19 time period, age, sex, stage, race and ethnicity, smoking status, BMI, Charlson Comorbidity Index, and healthcare system.

All analyses were performed using SAS^®^ Software version 9.4 (SAS Institute Inc., Cary, North Carolina, USA) and STATA^®^ Statistical Software version 18.0 (StataCorp. 2019. Stata Statistical Software: Release 16. College Station, TX, USA: StataCorp LLC).

## Results

**Figure 1:**
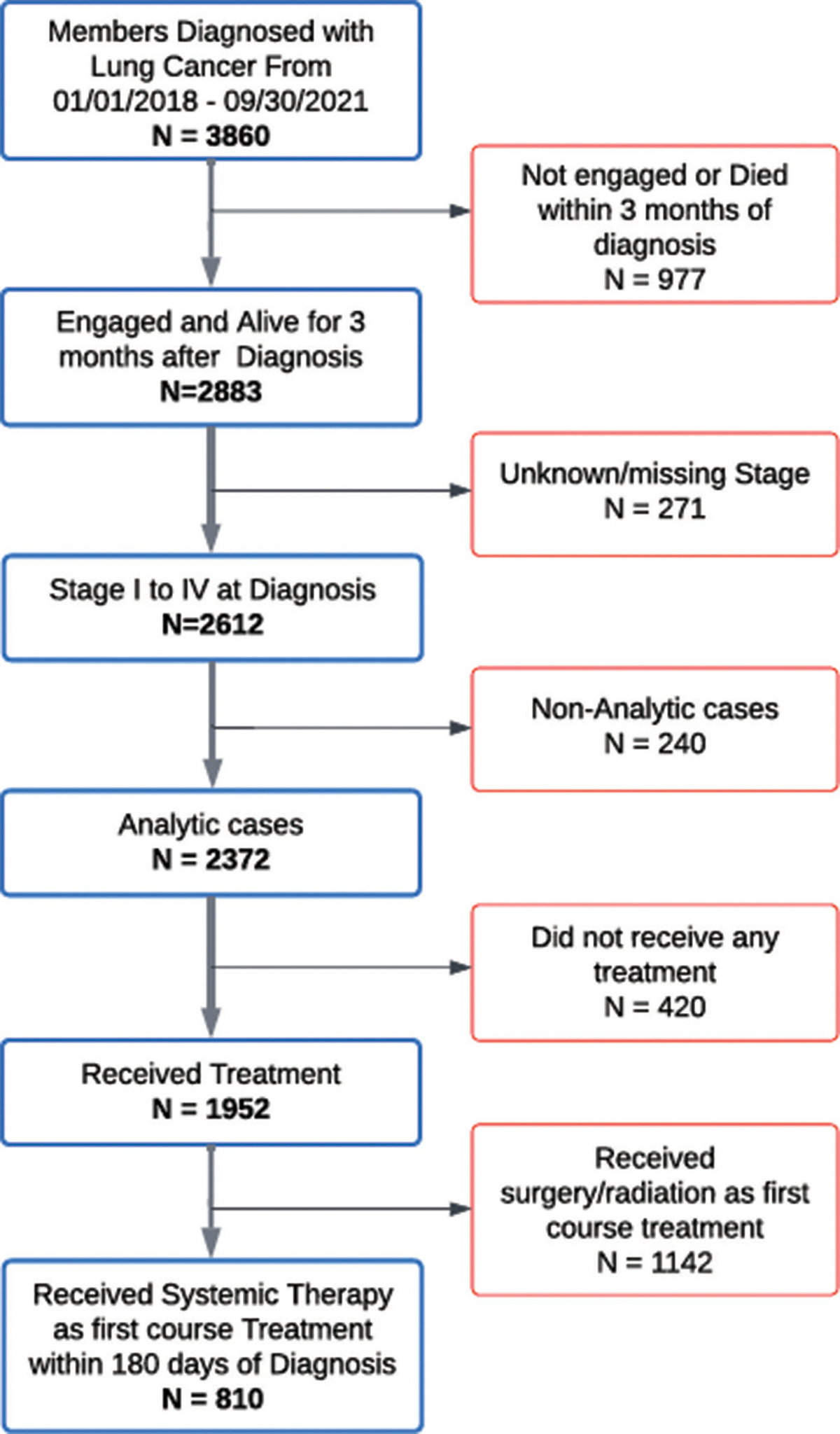
Flow diagram for patients included in this study

We excluded 1142 patients who received surgery or radiation therapy as the first course of treatment [Supplementary Table 1]. After applying the inclusion and exclusion criteria, our study cohort contained 810 unique individuals, of whom 460 were diagnosed during the pre-COVID-19 period (56.8%), 61 during the early COVID-19 period (7.5%), and 289 during the late COVID-19 period [35.7%; Figure 1 and Table 1]. A detailed flow diagram showing the number of participants included/excluded by COVID-19 time period and stage at diagnosis is provided in Supplementary Figure 1.

The monthly average number of patients diagnosed with lung cancer and treated with systemic therapy was 17.0 during the pre-COVID-19 period, which decreased to 15.3 during the early COVID-19 period and then increased to 19.3 during the late COVID-19 period. The patients diagnosed with lung cancer during the early COVID-19 period were more likely to be in the most deprived quintile of the Yost Index (27%) than those diagnosed in the pre- and late COVID-19 periods (21% and 23%, respectively). Similarly, the patients diagnosed in the early COVID-19 period were less likely to be in the top two most affluent quintiles of the YOST Index (32%) than those diagnosed in the pre- and late COVID-19 periods (39% and 46%, respectively). We also observed that some healthcare systems had larger reductions in cancer diagnoses during the early COVID-19 period. For example, the proportion of cancer diagnoses at healthcare system 1 decreased by 44% from the pre-to early COVID-19 periods (27% in the pre-period compared to 15% in the early period), then nearly returned to pre-COVID-19 levels by the late COVID-19 period. We also observed that more patients were diagnosed with late-stage lung cancer during the early COVID-19 period (82%) compared to the pre- and late COVID-19 periods (75% and 74%, respectively).

**Figure 2:**
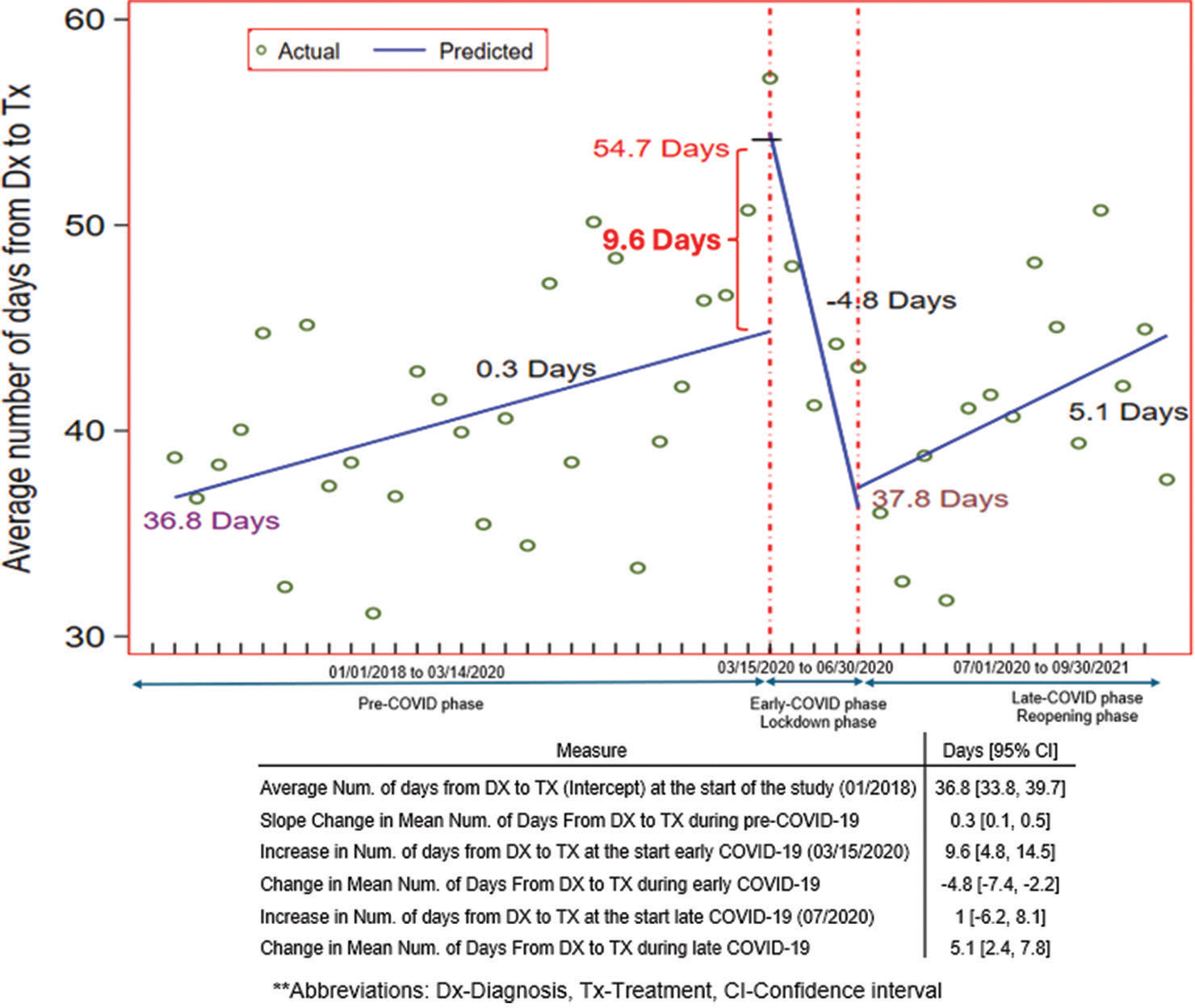
Trends and changes in the average number of days from diagnosis to first course systemic therapy by month

The average time from lung cancer diagnosis to initiating systemic therapy at the start of the pre-COVID-19 period was 36.8 days (95% confidence interval [CI]: 33.8–39.7), with a linear time trend indicating a 0.3-day increase (95% CI: 0.1–0.5) in the time to treatment for each month during the pre-COVID-19 period [Figure 2]. At the start of the early COVID-19 period, there was a 9.6-day (95% CI: 4.8–14.5, *P* = 0.0) increase from diagnosis to treatment compared to the last month of the pre-COVID-19 period, with a linear time trend showing a 4.8-day decrease (95% CI: −7.4–−2.2; *P* < 0.01) in the time to treatment for each month in the early COVID-19 period. In the 1^st^ month of the late COVID-19 period, there was a 0.3-day increase in the time to treatment (95% CI: 0.07–0.5; *P* = 0.01) compared to the last month of the early COVID-19 period, with a linear time trend showing that the average time to treatment increased by 5.1 days (95% CI: 2.4–7.8; *P* < 0.01) for each month during the late COVID-19 time period. Comparing the time to treatment from the 1^st^ month of the early COVID-19 period to the 1^st^ month of the late COVID-19 period, we found a nonstatistically significant increase of 1.0 days (95% CI: −6.2–8.1: *P* = 0.79) in the time to treatment.

**Figure 3:**
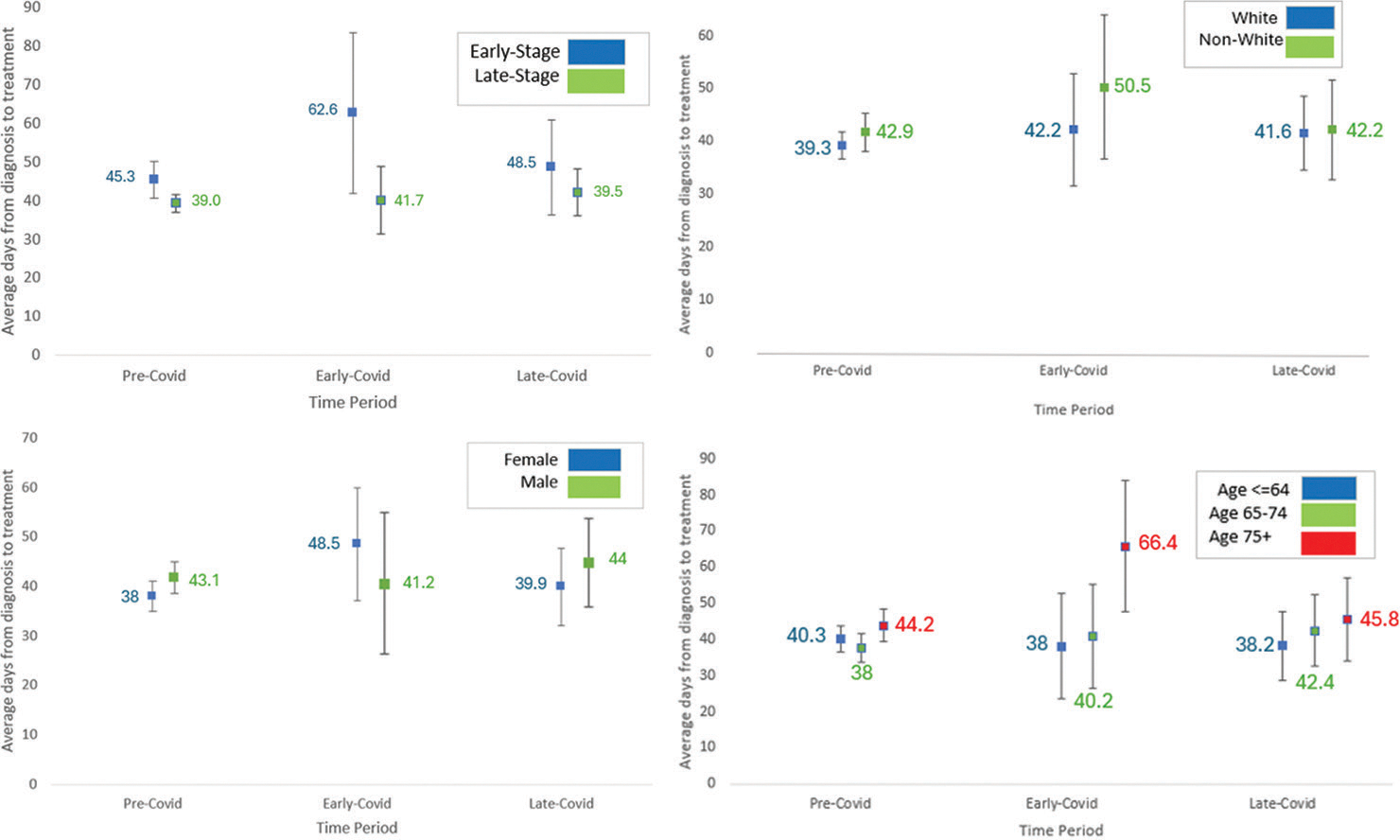
Unadjusted linear model of average number of days from lung cancer diagnosis to initiation of systemic therapy by COVID-19 time periods. (a) Stage at diagnosis: The interaction term COVID-19 time by stage is not statistically significant (*P* = 0.39), (b) Race: The interaction term COVID-19 time by race is not statistically significant (*P* = 0.54), (c) Sex: The interaction term COVID-19 time by sex is not statistically significant (*P* = 0.08), (d) Age groups: The interaction term COVID-19 time by age group is not statistically significant (*P* = 0.09)

Results stratified by stage at the time of diagnosis showed that the patients diagnosed with early-stage lung cancer had an average increase of 17.3 days (95% CI: 1.3–33.4) in the time to treatment between the pre- and early COVID-19 periods, whereas the patients diagnosed with late-stage lung cancer had a nonsignificant increase of 2.7 days (95% CI: −4.2–9.6 days) in the time to treatment between the pre- and early COVID-19 periods [Figure 3]. The delay in initiating treatment was more pronounced among the patients diagnosed with early-stage lung cancer.

Results stratified by race showed a non-significant 7.6-day increase (95% CI: −3.3–18.5) for non-White patients between the pre- and early COVID-19 periods, and a non-significant 2.9-day increase (95% CI: −5.2–10.9 days) for white patients. Results stratified by gender showed a 10.5-day increase (95% CI: 2.1–18.8 days) in the time to treatment for female patients between the pre- and early COVID-19 periods, whereas males showed a nonsignificant 1.9-day decrease (95% CI: −2.9–6.7 days) between these two periods. Results stratified by age showed that the older patients (75 years and older) had a 22.2-day increase (95% CI: 8.6–35.8 days) in the time to treatment between the pre-and early COVID-19 periods, whereas the patients aged 65–74 had a non-significant 2.2-day increase (95% CI: −7.5–11.9 days) and patients 64 and under had a nonsignificant decrease of 2.3 days (95% CI: −13.3–8.6 days).

Multivariable linear regression analyses showed a nonsignificant 5-day (95% CI: −1.4–11.1 days) increase in the time to initiating systemic therapy during the early COVID-19 period compared to the pre-COVID-19 period [Supplementary Figure 2]. We found a 7.3-day (95% CI: 3.0–11.7 days) increase in treatment initiation among the patients aged 75 or older compared to younger patients aged 35–64 years. The patients diagnosed with early-stage cancer had a longer time to therapy initiation (7.8 days; 95% CI: 4–11.5 days) compared to those diagnosed with late-stage cancer.

## Discussion

In a cohort of patients diagnosed with primary lung cancer, we estimated a 9.6-day increase from diagnosis to initiation of systemic therapy at the onset of the COVID-19 shutdown period. However, this delay was quickly abated in the months following the shutdown period, highlighting the ability of the healthcare systems to respond to treatment disruptions swiftly. In contrast to our findings, previous studies have not identified delays in the timeliness of receiving systemic therapy for lung cancer among patients diagnosed between 2019 and 2021.^[35,36]^ However, our findings show the specific impact of COVID-19 on systemic therapy initiation for lung cancer,^[11,12,22,35]^ whereas other studies have examined delays in lung cancer screening,^[11]^ delays in diagnostic testing,^[12,22]^ or delays in surgical treatment^[35]^ for lung cancer.

The 9.6-day increase in the time to initiating systemic therapy during the early COVID-19 period warrants careful evaluation in the context of existing clinical guidelines and research. We found that the time to treatment increased to 55 days at the onset of the COVID-19 pandemic. One previous study reported that a delay of over 6 weeks was associated with a 17% higher mortality rate in patients with non-small cell lung cancer,^[37]^ and another study reported lower 1- and 5-year overall survival rates when the time to treatment was >50 days.^[38]^ In addition, delayed treatment may contribute to higher symptom burden and psychological distress, further affecting patient well-being.^[37]^ Further research, including comparisons with established benchmarks and relevant clinical data, is essential to determine whether this delay substantially impacts patient outcomes. We did not find a significant difference in the number of days from diagnosis to treatment between the start of the early COVID-19 period and the start of the late COVID-19 period (36.8 days vs. 37.8 days). The 5.1-day increase during the late COVID-19 period may reflect natural fluctuations as the healthcare system caught up with treating patients before returning to pre-COVID-19 rates.

Our findings suggest that the early-stage cancer patients had greater treatment delays compared to those with late-stage cancer, likely due to the prioritization of advanced-stage cancer, as reported in prior studies.^[21]^ The prioritization of late-stage cases often necessitates urgent interventions due to advanced disease progression.^[39]^ In addition, challenges during the pandemic, such as modifications to elective surgery schedules, relocation of healthcare resources, shortages of systemic therapy medications, and disruptions in diagnostic pathways, may have disproportionately impacted systemic therapy initiation among patients diagnosed with early-stage cancer.^[4–6,23,39]^

We recognize that the proportion of late-stage patients excluded due to not receiving a first course of systemic therapy appears disproportionately lower in the late COVID-19 period. Patients with late-stage lung cancer were less likely to receive systemic treatment, which may be a result of the combination of clinical considerations and potential lingering effects of COVID-19. Because fewer late-stage patients received systemic therapy, our results may not have fully captured the extent of their delay, or treatment decisions may have been adjusted to prioritize overall health rather than an aggressive treatment approach, which may have resulted in an underestimation of the treatment delays for this subgroup.

Previous studies have suggested that the proportion of patients receiving lung cancer treatment did not decline during the COVID-19 shutdown.^[39,40]^ Tarawneh *et al*. and Patt *et al*. found a decrease in the number of cancer diagnoses and advanced-stage lung cancer cases during the early COVID-19 period, which is similar to our findings.^[19,22]^ However, the COVID-19 shutdown did lead to significant reductions in preventive care, with one study estimating a more than a 50% reduction in lung cancer screening during this period.^[22]^ A systematic review conducted by Muka *et al*. found that delays in treatment and screening during the early COVID-19 period were caused by decreased access, changes to treatment recommendations, and appointment cancellations.^[41]^ Powell *et al*. examined treatment trends using claims data between 2019 and 2021; however, they could not analyze trends of systemic therapy because of a lack of data availability in claims data, whereas our rich data resource allowed us to examine detailed systemic therapy treatment data.^[39]^

According to the National Comprehensive Cancer Network, systemic therapy is indicated for patients with late-stage cancers. However, nearly 25% (*n* = 201) of patients in our cohort diagnosed with early-stage cancer received systemic therapy as the first course of treatment. A chart review of a subset of these patients revealed that they were either receiving palliative care, had multiple cancers, other chronic conditions, or were not able to receive surgery, which agrees with guideline-recommended care for patients with early-stage lung cancer.

Our stratified results showed that the average time to treatment was higher among those 75 years and older compared to those aged 74 and under. We also found that the time to treatment among the female patients was higher than among the male patients during the early COVID-19 period. Mangone *et al*. analyzed the incidence of cancers during the COVID-19 pandemic and found a significant increase in lung cancer diagnoses among females.^[42]^ We also found that a higher number of female patients were diagnosed with lung cancer during the early COVID-19 period compared to male patients; however, Mangone *et al*. did not examine delays in receiving treatment during the COVID-19 pandemic.^[42]^ Previous research has suggested that older adults may experience delays due to more extensive pretreatment evaluations or non-cancer comorbid burden.^[43]^ While sex-based differences in treatment initiation have been linked to variations in symptom presentation and provider recommendations,^[44]^ a recent study on patients with Stage IV lung cancer did not find a difference between males and females in receiving systemic therapy.^[29]^ In addition, institutional policies, such as prioritizing certain patient groups during resource-constrained periods, particularly during the early COVID-19 period, could have inadvertently contributed to these disparities.^[14,15]^

Prior studies have examined the impact of treatment timing on survival and focused on assessing treatment delays as bivariate outcomes; however, they have not quantified the number of days.^[12,45–49]^ However, prior studies that evaluated delays in lung cancer screening, diagnosis, and treatment due to COVID-19 disruptions did not examine how quickly healthcare systems recovered from the delays because of short follow-up times.^[11,12,22,35,39]^ Due to the longer follow-up period in the present study, we were able to examine how quickly the healthcare systems recovered from such COVID-19 disruptions.

In addition to disruptions in healthcare access and alterations in treatment patterns affecting the timeliness of receiving systemic therapy, the early COVID-19 period was also marked by a severe shortage of essential chemotherapy drugs.^[4–6]^ These drug shortages impacted as many as 90% of hospital systems across the United States during the early stage of the COVID-19 pandemic.^[50]^ Treatment delays caused by drug shortages highlight the need for policies that optimize resource allocation during supply chain disruptions to ensure continuity of care in future emergencies. These policies may include establishing systemic cancer treatments as essential services and investing in telemedicine infrastructure to mitigate treatment delays.

### Limitations

The limitations of this study include that this was an observational study investigating patterns of treatment. In addition, our ITS methods used data aggregated to a monthly level. Thus, we could not make assumptions about individual-level outcomes. Future research using patient-level data could help disentangle these effects by incorporating other methods to adjust for individual-level confounders. Although our cohort was recruited from healthcare centers in various locations, our results may not be generalizable to racially and ethnically diverse populations. In addition, our cohort was continuously engaged with a healthcare system during the study period; individuals who become uninsured following a lung cancer diagnosis may have faced other barriers to access, leading to longer delays in cancer treatment, which may have also affected the generalizability of our findings; future research is needed to examine disparities in treatment delays among uninsured and underinsured populations. Another limitation is that given that the end of our study period was December 2021, we could not assess variations in survival outcomes by time to treatment. Future studies should evaluate the clinical impact of treatment delays. Moreover, the small sample size during the early COVID-19 period limited the precision of our findings. Finally, our analysis did not account for competing risks, such as death before receiving treatment.

## Conclusion

Our results shown a 9.6-day increase from lung cancer diagnosis to initiation of systemic therapy immediately after the start of the COVID-19 shutdown period. However, the time to treatment quickly recovered to pre-COVID-19 rates during the post-COVID-19 period. As healthcare systems adapt to post-pandemic realities, prioritizing policies and interventions that safeguard timely cancer treatment is essential to protect vulnerable populations. Policymakers should implement strategies that can withstand disruptions to patient care by designating systemic therapy as an essential service, strengthening supply chain resilience, and expanding telemedicine infrastructure to minimize treatment delays. Healthcare administrators and oncologists must strengthen clinical governance policies, ensuring a more resilient, efficient, and equitable healthcare system. Further research is needed to assess the long-term impacts of delayed systemic therapy on lung cancer outcomes, including survival and costs, and to evaluate effective strategies for mitigating treatment delays.

## Supplementary Material

1

## Figures and Tables

**Table 1:** Demographic characteristics of patients diagnosed with lung cancer receiving systemic therapy as first course of treatment (2018–2021)

	Pre-COVID-19 (*n*=460) (January 1, 2018–March 14, 2020), *n* (%)	Early-COVID-19 (*n*=61) (March 15, 2020–June 30, 2020), *n* (%)	Late-COVID-19 (*n*=289) (July 1, 2020–September 30, 2021), *n* (%)	*P*

Age groups (years)				0.83
35–64	164 (36)	20 (33)	101 (35)	
65–74	168 (36)	27 (44)	110 (38)	
75–89	128 (28)	14 (23)	78 (27)	
Sex				0.38
Female	233 (51)	36 (59)	156(54)	
Male	227 (49)	25 (41)	133 (46)	
Race and ethnicity				0.72
Asian	29 (6)	5 (8)	21 (7)	
Black	61 (13)	10 (16)	47 (16)	
Native Hawaiian/Pacific Islander	24 (5)	6 (10)	13 (5)	
Hispanic	22 (5)	<6	9 (3)	
Unknown/Other race	19 (4)	<6	13 (5)	
White	305 (67)	37 (60)	186(64)	
Stage at diagnosis				0.40
Early (AJCC Stage I-IIIA)	114(25)	11 (18)	76 (26)	
Late (AJCC Stage IIIB-IV)	346 (75)	50 (82)	213 (74)	
BMI at diagnosis (kg/m^2^)				0.59
<25	171 (37)	26 (43)	121 (42)	
25–29	166 (36)	23 (38)	97 (34)	
30 or more	123 (27)	12 (20)	71 (24)	
Charlson Comorbidity Index				0.20
<3 conditions	22 (5)	0 (0)	15 (5)	
≥3 conditions	438 (95)	61 (100)	274 (95)	
Smoking status at diagnosis				0.31
Currently smokes	175 (38)	25 (41)	102 (35)	
Formerly smoked	215 (47)	30 (49)	130 (45)	
Unknown smoking history	70 (15)	6 (10)	57 (20)	
YOST State Quintile				0.023
1 (most deprived)	80 (21)	14 (27)	60 (23)	
2	95 (25)	6 (11)	42 (15)	
3	59(15)	15 (30)	44 (16)	
4	79 (21)	7 (13)	60 (23)	
5 (Most affluent)	68 (18)	10 (19)	60 (23)	
Healthcare system				0.017
1	124 (27)	9 (15)	68 (23)	
2	73 (16)	14 (23)	42 (15)	
3	79 (17)	6 (10)	34 (12)	
4	184(40)	32 (52)	145 (50)	

AJCC: American Joint Committee on Cancer, BMI: Body mass index

## Data Availability

The data generated for this study are from electronic health records, contain individual-level data like PHI, and contain intellectual properties that cannot be released without an individual’s consent.
