# Supplementary material for: Impact of COVID-19 on Timeliness of Receiving Systemic Therapy for Patients Diagnosed with Lung Cancer
Source: J Cancer Res Pract. Author manuscript; Available in PMC 2025 Dec 25. (PMC12728826; doi:10.4103/ejcrp.ejcrp-d-25-00002)
Supplement: 1 [file NIHMS2125372-supplement-1.pdf]

**Supplementary Table 1: Demographic characteristics of patients diagnosed with lung cancer receiving surgery or radiation as first course of treatment (2018–2021)**

| | Pre-COVID-19 ($n$=712) (January 1, 2018–March 14, 2020), $n$ (%) | Early-COVID-19 ($n$=75) (March 15, 2020–June 30, 2020), $n$ (%) | Late-COVID-19 ($n$=355) (June 1, 2020–September 30, 2021), $n$ (%) | $P$ |
|---|---|---|---|---|
| Age at the time of diagnosis | | | | 0.62 |
| 35–64 | 221 (31.0) | 24 (32.0) | 97 (27.3) | |
| 65–74 | 275 (38.6) | 31 (41.3) | 139 (39.2) | |
| 75–89 | 216 (30.3) | 20 (26.7) | 119 (33.5) | |
| Sex | | | | <0.01 |
| Female | 408 (57.3) | 52 (69.3) | 177 (49.9) | |
| Male | 304 (42.7) | 23 (30.7) | 178 (50.1) | |
| Race and ethnicity | | | | 0.36 |
| Asian | 40 (5.6) | 4 (5.3) | 10 (2.8) | |
| Black | 78 (11.0) | 6 (8.0) | 45 (12.7) | |
| Hispanic | 34 (4.8) | 5 (6.7) | 22 (6.2) | |
| Native Hawaiian/Pacific Islander | 23 (3.2) | 3 (4.0) | 6 (1.7) | |
| Unknown/Other race | 27 (3.8) | 4 (5.3) | 9 (2.5) | |
| White | 510 (71.6) | 53 (70.7) | 263 (74.1) | |
| Stage at diagnosis | | | | 0.01 |
| Early (AJCC stage I-IIIA) | 562 (78.9) | 59 (78.7) | 306 (86.2) | |
| Late (AJCC stage IIIB-IV) | 150 (21.1) | 16 (21.3) | 49 (13.8) | |
| BMI at diagnosis (kg/m$^2$) | | | | 0.52 |
| <25 | 250 (35.1) | 28 (37.3) | 140 (39.4) | |
| 25–29 | 251 (35.3) | 26 (34.7) | 127 (35.8) | |
| 30 or more | 211 (29.6) | 21 (28.0) | 88 (24.8) | |
| Charlson Comorbidity Index | | | | 0.56 |
| <3 conditions | 84 (11.8) | 6 (8.0) | 44 (12.4) | |
| ≥3 conditions | 628 (88.2) | 69 (92.0) | 311 (87.6) | |
| Smoking status at diagnosis | | | | 0.84 |
| Currently smokes | 191 (26.8) | 20 (26.7) | 93 (26.2) | |
| Formerly smoked | 394 (55.3) | 37 (49.3) | 196 (55.2) | |
| Never smoked | 118 (16.6) | 16 (21.3) | 59 (16.6) | |
| Unknown | 9 (1.3) | 2 (2.7) | 7 (2.0) | |
| YOST State Quintile | | | | 0.38 |
| 1 (Most deprived) | 115 (19.2) | 11 (15.9) | 75 (23.4) | |
| 2 | 122 (20.4) | 19 (27.5) | 60 (18.8) | |
| 3 | 123 (20.6) | 14 (20.3) | 52 (16.2) | |
| 4 | 124 (20.7) | 12 (17.4) | 61 (19.1) | |
| 5 (Most affluent) | 114 (19.1) | 13 (18.8) | 72 (22.5) | |
| Healthcare system | | | | <0.01 |
| 1 | 284 (39.9) | 45 (60.0) | 157 (44.2) | |
| 2 | 80 (11.2) | 6 (8.0) | 11 (3.1) | |
| 3 | 94 (13.2) | 9 (12.0) | 33 (9.3) | |
| 4 | 254 (35.7) | 15 (20.0) | 154 (43.4) | |
| First course surgery | | | | 0.08 |
| Yes | 432 (60.7) | 44 (58.7) | 239 (67.3) | |
| No | 280 (39.3) | 31 (41.3) | 116 (32.7) | |
| First course radiation therapy | | | | 0.08 |
| Yes | 280 (39.3) | 31 (41.3) | 116 (32.7) | |
| No | 432 (60.7) | 44 (58.7) | 239 (67.3) | |

AJCC: American Joint Committee on Cancer, BMI: Body mass index

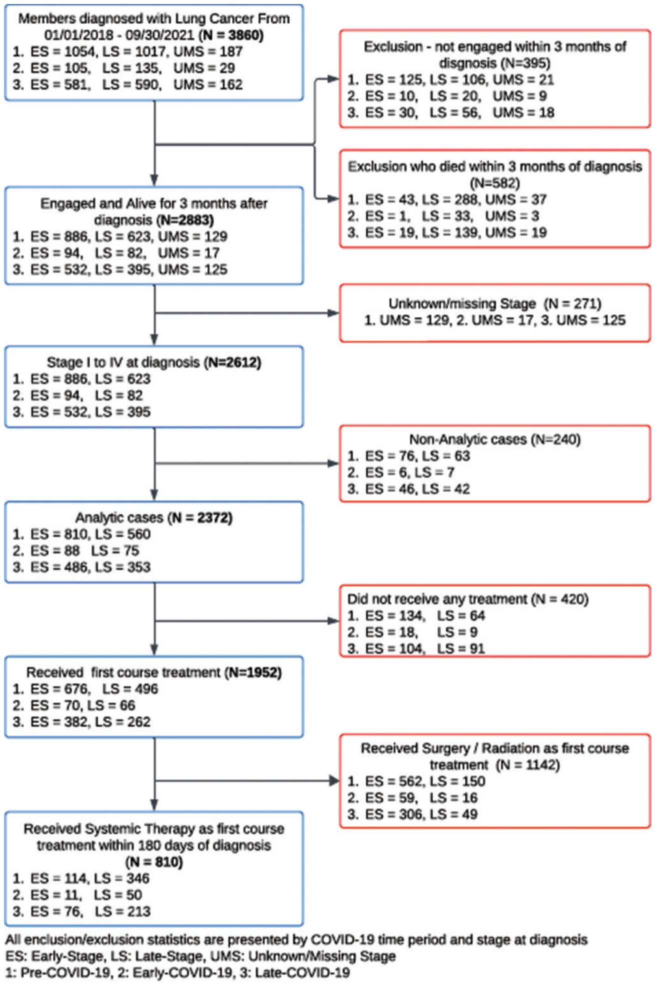


**Supplementary Figure 1:** Detailed flow diagram of inclusion-exclusion by COVID-19 time periods and stage at the time of diagnosis for the study cohort

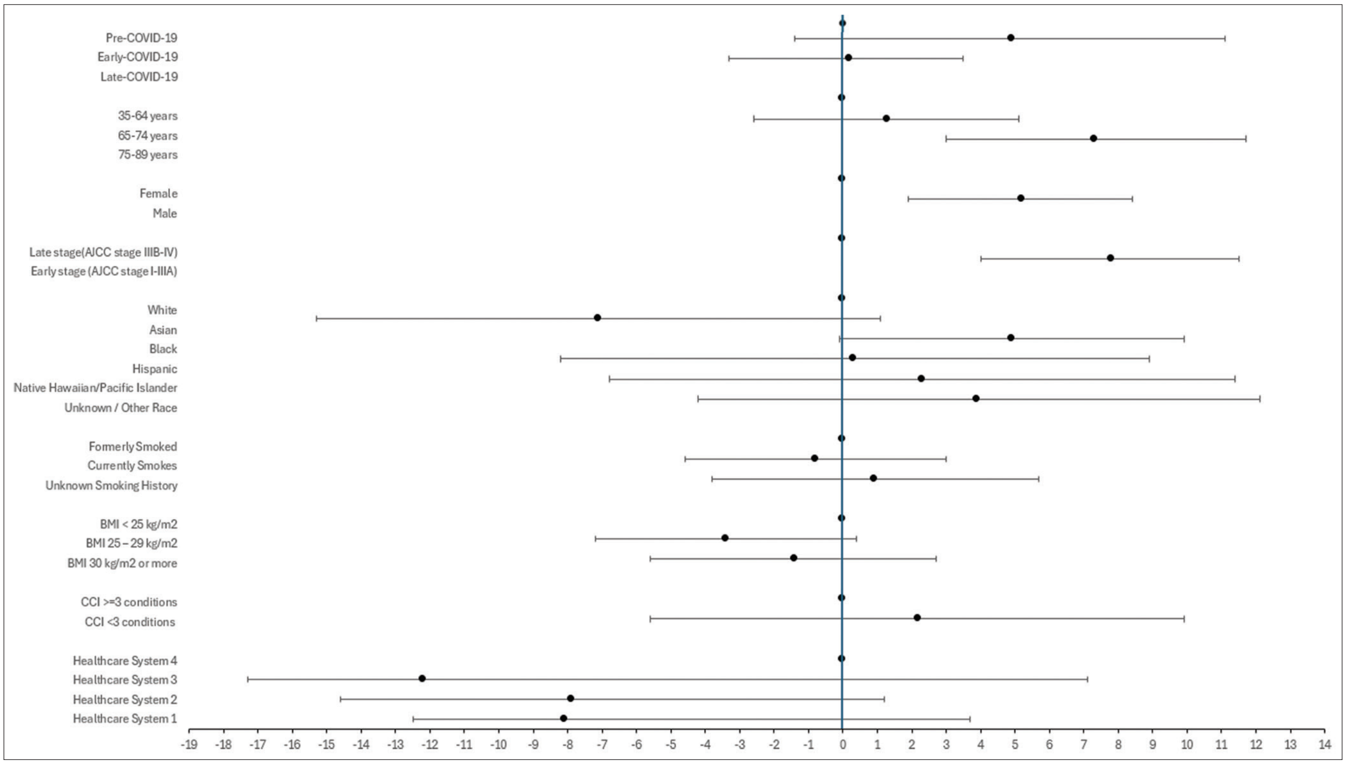


**Supplementary Figure 2:** Multivariable linear regression estimates
